# Client-provider interaction: understanding client experience with family planning service providers through the mystery client approach in India

**DOI:** 10.1080/26410397.2020.1822492

**Published:** 2020-10-15

**Authors:** Ankita Shukla, Rajib Acharya, Abhishek Kumar, Arupendra Mozumdar, Kumudha Aruldas, Niranjan Saggurti

**Affiliations:** a Program Officer, Population Council, New Delhi, India; b Associate II, Population Council, New Delhi, India; d Senior Program Officer, Population Council, New Delhi, India; e Implementation Science Coordinator, Deworm3 Study, Christian Medical College, Vellore, India; f Director, Population Council, New Delhi, India

**Keywords:** quality of care, providers’ preferences, family planning, privacy, client-provider interaction

## Abstract

The benefits of employing a rights-based approach in family planning (FP) programmes have made the client’s rights to informed choices and quality care an essential part of any such programme. client-provider interaction is one of the critical components of the quality of care (QoC) framework of FP. While several studies have assessed QoC in FP services in India, very few have focused on the in-depth assessment of the interaction between the client and the provider during service delivery. The present study used the mystery client approach to assess the quality of interactions between clients and FP service providers in two of the most populous states of India: Bihar and Uttar Pradesh (UP). Findings highlighted that the providers spent very little time with the clients, gave them information on only one or two FP methods, and rarely talked about possible side-effects of the methods. Furthermore, the providers seemed hesitant to suggest any FP method other than condoms to newly married women. This study concluded that despite being a government priority, the quality of client-provider interaction in these two states was extremely poor.

## Résumé

Les avantages de l’emploi d’une approche fondée sur les droits dans les programmes de planification familiale ont fait du droit des clients à un choix éclairé et à des soins de qualité une partie essentielle de tout programme de ce type. L’interaction entre les clients et les prestataires est l’un des volets essentiels du cadre sur la qualité des soins dans la planification familiale. Si plusieurs études ont évalué la qualité des soins dans les services de planification familiale en Inde, rares sont celles qui se sont centrées sur l’évaluation approfondie de l’interaction entre le client et le prestataire pendant la réalisation des services. La présente étude a utilisé la technique du client mystère pour juger de la qualité des interactions entre clientes et prestataires de services de planification familiale dans deux des États les plus peuplés de l’Inde: le Bihar et l’Uttar Pradesh. Les conclusions ont révélé que les prestataires passaient très peu de temps avec les clientes, leur donnaient des informations sur seulement une ou deux méthodes de planification familiale et parlaient rarement des possibles effets secondaires des contraceptifs. De plus, les prestataires semblaient hésiter à suggérer une méthode autre que les préservatifs aux jeunes mariées. Cette étude a conclu que bien qu’il s’agisse d’une priorité gouvernementale, la qualité de l’interaction cliente-prestataire dans ces deux États étaient extrêmement faible.

## Resumen

Los beneficios de emplear un enfoque basado en los derechos en los programas de planificación familiar (PF) implican que los derechos de las usuarias a tomar decisiones informadas y recibir atención de calidad son una parte esencial de este tipo de programa. La interacción usuaria-prestador/a de servicios es uno de los componentes fundamentales del marco de calidad de la atención (CdA) de la planificación familiar. Aunque varios estudios han evaluado la CdA en servicios de PF en India, muy pocos se han enfocado en la evaluación detallada de la interacción entre la usuaria y el/la prestador/a de servicios durante la prestación de servicios. El presente estudio utilizó el enfoque de usuaria sorpresa para evaluar la calidad de las interacciones entre usuarias y prestadores de servicios de PF en dos de los estados más poblados de India: Bihar y Uttar Pradesh (UP). Los hallazgos destacaron que los prestadores de servicios dedicaron muy poco tiempo a cada usuaria, les proporcionaron información sobre uno o dos métodos de PF solamente, y rara vez hablaron sobre los posibles efectos secundarios de los métodos. Más aún, los prestadores de servicios se mostraron reacios a sugerir otros métodos de PF además de los condones a mujeres recién casadas. Este estudio concluyó que, a pesar de ser prioridad del gobierno, la calidad de la interacción entre usuaria y prestador/a de servicios en estos dos estados era muy mala.

## Introduction

Inclusion of the rights-based approach in family planning (FP) programmes has been endorsed by several international communities and conventions for over 50 years.^1–5^ Recent commitments, made at the London Summit of Family Planning, have renewed the emphasis on protecting and fulfilling a client’s rights to achieve the set goals.^6^ In response to this, FP programmes across the world have been focusing on providing quality services to clients by giving complete information along with appropriate and respectful counselling based on the needs of clients.^7^

Client-provider interaction is one of the essential components in the FP quality of care (QoC) framework^8,9^ and critical to ensuring that clients have the rights as well as information to make an informed choice. It is argued that providing information alone to clients is not enough; rather, programmes should focus on two-way communication between providers and clients. If the information provided to clients is adequate and according to their needs, clients are more likely to adopt and continue using FP methods.^10,11^

The quality of client-provider interaction includes multiple components such as maintaining privacy, informing clients on the full range of contraceptives available in the health system, providing information on side effects of the suggested methods and their management, follow-up care, options of switching from one method to another, giving appropriate time and attention, and addressing and respecting the client’s concerns and choices.^9,12^ Service providers are expected to create an environment where clients feel respected, and enable them to share concerns with service providers without hesitation.^13,14^ Creating such an environment can positively influence potential clients towards using FP methods and their decision to continue with the method, resolve any problems and, if needed, help them switch to another method.^15,16^ However, past studies have documented that service providers show negative attitudes towards the clients while rendering FP services.^17^ In low- and middle-income countries, including India, poor and uneducated women are often made to adopt FP methods without being involved in the decision-making process. The selection of the method is often not the client's informed decision, but influenced by FP providers or family members.^18–20^

In India, studies on client-provider interaction have largely focused on the utilisation of maternal and child healthcare services.^21–23^ These studies highlighted poor consideration by the providers towards clients’ privacy as well as not encouraging clients to ask questions and actively participate in antenatal care services.^23^ Only a few studies in India assessed the extent of information given by FP providers to women who wanted to adopt contraceptive methods. One study, based on a large scale household survey, found that informed choice related to the chosen FP method was very low.^24^ Another study, based on exit interviews of FP clients, found that information exchange between providers and clients was limited at the time of choosing the method, resulting in a small proportion of women receiving their method of choice.^20^ One of the limitations of the study was possible bias on the part of the clients while reporting on their interaction with the provider. Another way to study client-provider interaction is through direct observation of the interaction. In such situations, the Hawthorne effect, i.e. change in the natural behaviour of the providers in response to their awareness of being observed, may bias the outcome. Moreover, a study of client-provider interaction through direct observation may make the client uncomfortable due to the presence of an outside person, and threaten her privacy.

The simulated or mystery client (MC) approach is a balanced way to examine the client-provider interaction in FP services, and has been adopted by a few studies to assess client experiences.^25–27^ In this approach, trained women acting as FP clients visit health facilities to generate real-time data on client-provider interaction free from recall limitations, personal bias and the Hawthorne effect. The present study also used this approach to assess the quality of interaction between clients and FP service providers. Its findings will be useful for policymakers and programme managers in improving QoC of FP services, including support for continuation of methods adopted by clients.

## Materials and methods

### Quality components of client-provider interactions

**Figure 1.**
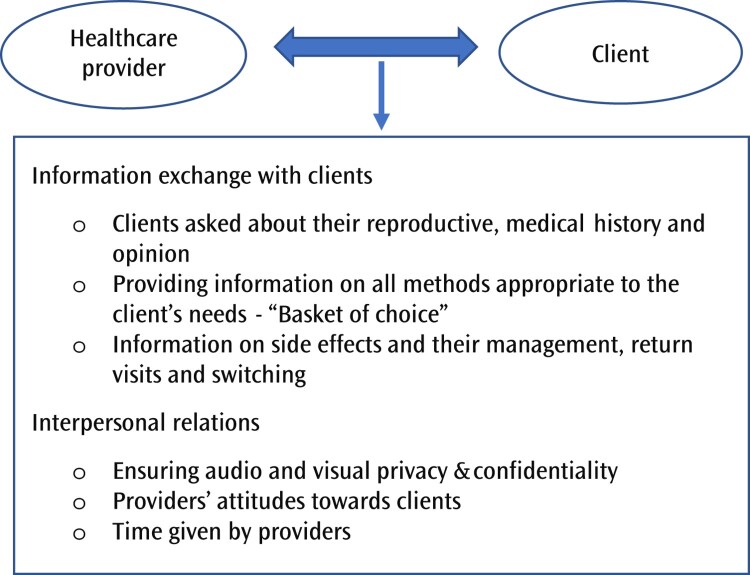
Quality of care components in client-provider interaction.

This study assessed two important domains of QoC during the delivery of FP services: information exchange with clients, and interpersonal relations. The component of continuity of care included by Jain and Hardee^9^ in their revised QoC framework could not be assessed because the data were collected from MCs who, in reality, did not finally adopt any method as they were only posing as clients. Finally, the component of time given by the provider was included under the domain of interpersonal relations as we hypothesised that the longer the interaction, the better the quality of client-provider interaction. Specific components of these two domains of QoC are presented in Figure 1.

### Study area

This qualitative study was conducted in two of the most populous states in India: Bihar and Uttar Pradesh (UP). Both these states reported low modern contraceptive prevalence rate (mCPR) and skewed method mix. In fact, the contribution of the female sterilisation method to overall mCPR was 89% and 55% in Bihar and UP respectively.^28^ Available data show that QoC of FP services was poor in both these states. For example, the method information index (MII), an indicator of QoC of FP services, was found to be very low in these states.^24^ A large proportion of current users of FP reported not being informed on side-effects or their management.^28^

From each state, two districts were selected: one district with mCPR higher than the state average and with a balanced method mix, and another district with mCPR lower than the state average and with a skewed method mix. From each district, two administrative blocks (a block is a defined subdistrict area) were selected based on the ratio of intrauterine contraceptive device (IUCD) to female sterilisation services provided in the public health facilities of the block (one block with a high ratio and one with a low ratio). For visits to public health facilities, one district hospital from each district and one Community Health Center or Primary Health Center from each block was selected. Since no formal list is available for private health facilities, a list of private facilities providing FP services in the study area was prepared by the field investigators and six facilities randomly selected from each district.

### Data collection

Data for this study were collected between 2017 and 2018 in the four selected districts of Bihar and UP using the MC approach. Each MC was given a specific scenario to act (Table 1). Seven women were engaged as MCs to visit FP service providers in the study area. Since very few men visit facilities for FP consultation it was decided not to use male MCs as they would stand out from the rest of the clients in the facility and risk being identified as not real clients. Women selected to play the role of MC were from the communities similar to the study areas, spoke the same dialect, and knew the local culture, including local dress and lifestyle. These qualities made them suitable to perform the role of MCs in the study area.

**Table 1.** Details of scripted mystery client scenarios.

	Scenario	Characteristics of MCs
1	*Newly married woman* A newly married woman aged 21 years who was living with her parents until now. Since she wishes to study further, she is considering delaying pregnancy. She recently started living with her husband and hence wants to use any FP method to delay childbirth for at least 1–2 years.	One young 21-year-old unmarried girl was selected to play the role of newly married women. The girl has a graduate degree.
2	*Women seeking advice for spacing between births* (a) A married woman aged 27 years who only has a one-year-old girl child. She wants some time to raise her daughter and wishes to use any method to delay the next birth.	Two married women aged 27 and 25 years were selected to play this role. They were educated and had their own children.
	(b) A 28-year-old married woman with two daughters aged 3 and 1 years. She wants to use a suitable modern FP method to delay the next birth.	This role was played by two married women aged 28 and 29 years. They were educated and had their own children.
3	*Woman seeking advice for limiting births* A 30-year-old woman who already has three children and does not want any more children. She is looking for a long-term/permanent method.	For this role two older married women were selected (age 30 and 35 years) who themselves had two children. The selected participants had completed their family size and personal experience of using family planning methods.

The training of MCs involved familiarising them with the allotted scenarios – their fictional age and education status, number of children, a false address, and a fictitious husband’s name and occupation, if asked by the provider. They were also trained on how to avoid any physical examination by the FP service provider if suggested during the visit. The MCs were told to let the provider talk uninterruptedly, but if the provider was not talkative, they were trained to probe and stretch the conversation further to collect more information. All MCs were asked to conclude their session by taking a free packet of condoms or oral/emergency contraceptive pills, if available, from the service provider before leaving the facility. Once all the MCs were confident in playing their respective roles, the research team conducted several mock trials of their visits to providers to test their readiness. All MCs were asked to meet the doctor who provides FP services in the selected facilities. Though counsellor and trained nurses are also involved in FP service provision, we chose to assess clients’ interactions with doctors only, as counsellors are not universally available in public facilities, and there are none in private facilities. Finally, because nurses are closer to the community, there is a higher risk of MCs’ identity disclosure.

For small qualitative studies, a minimum sample size of 12 is recommended to reach data saturation.^29,30^ Based on this guideline, the authors first decided to conduct at least 15 interviews in each district. With experience in the field and the amount of information available with each additional interview, it was decided to stop after a sample of 19 in each district.

In total, 76 visits were made by the MCs (Table 2). In all public and private facilities, MCs completed their visits without being identified by health staff. Following each visit, MCs were interviewed by trained research investigators using an interview guideline in their native language, Hindi, for an average of 40 minutes. The interview guideline was prepared based on the essential components of quality client-provider interaction (Figure 1) and pre-tested. Research investigators were trained by authors to interview the MCs. The investigators wrote the interviews verbatim in Hindi and the authors analysed the transcripts and summarised the results. Summaries from the interviews were then translated in English by the authors who are all fluent in both Hindi and English. To check that the original meaning did not change, summaries were translated and back-translated. The authors listened to the audio recording of all the interviews to check the quality and validity of the transcripts. Final transcripts were uploaded onto ATLAS.ti software and the authors analysed the transcripts and summarised the results.

**Table 2.** Distribution of mystery client visits by type of health facility.

		Playing the role of:			
Type of facility		A newly married woman	A woman seeking advice for spacing between births	A woman seeking advice for limiting births	Total
Public health facilities	District hospital	4	6	3	13
	PHC/CHC	7	12	6	25
Private clinics	At district and block	10	17	11	38
Total		21	35	20	76

### Analysis

The analysis was guided by a thematic approach. Initially, after reviewing the transcripts, based on the existing QoC framework, a set of codes was developed and categorised into three broad themes: quality of client’s experience at the reception desk, quality of information exchanged with clients, and quality of interpersonal relations. Codes were finalised through repeated discussions among the authors until a consensus was reached. The authors then recoded the transcripts using the final list of codes through ATLAS.ti software. A summary of results from the transcript analysis is presented, along with relevant quotations verbatim.

### Ethical considerations

Institutional Review Board (IRB) approval was obtained from the Population Council, New York, USA (protocol number 822 dated 10 July 2017). This project was approved by the Ministry of Health and Family Welfare, India, and the state Governments of Bihar and UP. The study also received required support and assistance from the chief medical officers – the highest health authority in a district – for conducting the study in their respective districts. All MCs were briefed on the study, its objectives, and its procedures. After clearly understanding the nature of the study, MCs were asked to sign written consent forms stating their willingness to participate in the research. Confidentiality and anonymity of both MCs and the providers were maintained by using codes to identify them during fieldwork and report writing.

## Results

### Quality of client experiences at health facility reception desk

Overall, MCs reported that the staff they met at the reception desks were polite. The average time spent at the reception desk was 2 minutes in private and 11 minutes in public facilities mostly because of higher client load in the public facilities. Across many public health facilities, there were no signs directing the clients to the room/point of service for FP, and therefore MCs often faced difficulties finding the room for FP services.

“The man at the reception desk told me to go to room no. 3 for FP services, but I could not find the doctor there. I looked around for some time…. I asked another employee *[staff at health facility]* …. then he told me the doctor's room would be room no. 5 *[not room no. 3]* …. where I found the doctor.” *(Public facility, Bihar, ID46)*

Staff at these facilities seemed overburdened and were sometimes reluctant to make any extra efforts to guide the women in finding the designated room for FP services or meeting the doctor. In private facilities MCs did not face these problems as private facilities are mostly small clinics led by one or two doctors. In public health facilities, tasks of the receptionist were mainly limited to doing the necessary paperwork.

On average, it took 15 minutes to meet with the doctor – 17 minutes in public facilities, and 12 minutes in private facilities. Moreover, in facilities with a heavy patient burden, the waiting time increased to an average of 22 minutes in public and 18 minutes in private health facilities.

### Quality of information exchange with clients

The findings discussed are related to: providers’ conversation with clients about their reproductive intentions; medical history; methods suggested by providers; use of information, education and communication (IEC) materials to explain the methods; information on side effects and their management; return visits; and switching to other methods.

#### Provider asked clients about their reproductive, medical history and opinion

In 55 out of the 76 visits, MCs were asked about their reproductive history. They were asked about their contraceptive history in 17 visits only. The medical history of MCs was rarely elicited. In only 27 visits were MCs asked about their opinion on the “prescribed” contraceptive method. There were instances where providers suggested a single method to the client without discussing her reproductive history, intention or needs.

“The provider asked me about my periods and [when she came to know that] I wanted to delay childbirth, … she did not discuss anything or gave me any choices, she directly wrote pills on the prescription.” *(Private facility, Bihar, ID64)*.

“When I went to see the doctor, she took my case-sheet and said ‘..taking pills or using an intrauterine contraceptive device (IUCD) will also be good for you’ … She did not give me time to ask any questions. She gave me my prescription back and asked to go … I will not say she was angry but was in a hurry – maybe due to the crowd of patients in front of her.” *(Public facility, UP, ID80)*

#### Providing information on all methods appropriate to the client’s needs – “basket-of-choice”

Findings were related to the types of methods suggested by the providers to: newly married women, those women who want to space between births, and those women who want to limit. On average, providers talked about three methods with clients in both public and private health facilities.

##### Contraceptives for newly married woman – delaying the first birth

Providers from both public and private facilities were hesitant to talk to newly married women about the range of contraceptive methods available. Though providers knew other methods, most of them suggested condoms and pills as the best methods for such clients.

“Provider talked about condoms, pills, and IUCD. When I asked, she also mentioned injectables but said you could not take injections as you didn’t have any children … . ‘With IUCD too you will face some problems. It is better to use pills or ask your husband to use condoms. Both will be suitable for you’.” *(Public facility, Bihar, ID41)*

“She did not talk to me much and directly prescribed pills. I had to ask if there were any other methods, only then she mentioned condoms, IUCD, and injections. But she said ‘injections and IUCD will not be good for you as you don’t have any children’.” *(Private facility, Bihar, ID64)*

There were some instances where providers had a negative attitude towards the use of pills by nulliparous women.

“The doctor said ‘ … there are many medicines, but those are harmful. The eggs that are formed after your periods are destroyed and you will have a problem in conceiving later. That’s why these methods are not good. [The] best that you can use, [is] condom, that will not cause any problem in the future’.” *(Private facility, UP, ID74)*

As newly wed women do not have any children, providers were cautious in recommending methods that may have side effects related to the menstrual cycles of women. One of the providers even advised the MC to have a child first, before thinking about using any method.

“The doctor said, ‘If you ask me, I will advise you to not use any method as no method is good for you. Lots of young women nowadays come for infertility treatment and you are saying that you want to delay childbirth? I think you should have a child first and then use any method’.” *(Private facility, UP, ID74)*

Only one provider talked about HIV and other sexually transmitted diseases (STDs) while interacting with a young newly married woman. There were only two instances where the provider appreciated the client for coming to the health facility just after marriage; and in five instances, MCs were asked about their method of choice among the contraceptive options available in that facility.

##### Contraceptives for spacing births

Irrespective of the type and level of the facility, most providers advised IUCD use to the MCs for spacing, sometimes even by discouraging other methods. However, a few providers tried to explain what the IUCD is, how it works, and its side effects, instead of just recommending the method.

“The doctor said “IUCD is best for you, once you have it inserted you will be free for a long time. If you choose injections or pills and miss a dose you will be at risk of conceiving. With IUCD you don’t have to worry about it’.” *(Private facility, Bihar, ID60)*

“The doctor told me that IUCD would be good in my situation … when I asked, ‘aren’t there other methods?’ then only, she [the doctor] said, ‘You can use Mala-N [a brand of contraceptive pills] too. Injectables are also available in the district hospital but I don’t advise them as they hamper the menstrual cycle’.” *(Public facility, UP, ID88)*

Other methods such as injectables, pills, and condoms were also suggested by the providers, but mostly as a second choice.

“She suggested that I should use Copper-T. I said ‘I don’t want to have Copper-T right away please tell me about other methods’. She said ‘then you can have injections’. I asked if it was safe? She said ‘yes, you can use it’. I said ‘I want something which does not cause any problems’. Then she talked about pills. She said ‘you can either take a pill daily or opt for injections, which you will have to take every three months or choose copper-T and be stress-free for 5–10 years’.” *(Public Facility, UP, ID78)*

Providers did not have in-depth discussions with MCs. For example, none of the MCs were informed about the recommended birth-interval and providers asked MCs about their method of choice in only 6 out of 35 MC visits.

##### Contraceptives for limiting births

Mystery clients who told the provider that they wanted to limit childbirth were predominantly advised by providers to opt for female sterilisation. This was reported in both public and private health facilities, especially in Bihar.

“She [the doctor] asked me to go for sterilisation. When I asked her about injectables, she said ‘it’s not available here’ … She did not even talk about pills in detail. … She said, ‘I don’t have knowledge about other [methods]; I’ll advise you to choose sterilization’ … whatever I asked after that she did not answer.” *(Public facility, Bihar, ID47)*

“At the very first, I was advised that if I didn’t want any more children then I should choose sterilisation. The doctor said, ‘There are two other methods you can use: IUCD and injection’ and then [the doctor] moved to the next patient.” *(Private facility, Bihar, ID27)*

In UP, especially in one district, there were slight variations in suggestions from the providers for limiting childbearing, which included pills, IUCDs, and injections apart from female sterilisation.

### Use of IEC materials

Posters and wall paintings on FP services and maternal and child healthcare were seen by MCs in all public health facilities but were not seen at private facilities. However, no specific IEC materials were used during their interaction with doctors. Only seven providers in Bihar and two in UP used samples/models while explaining the use of contraceptive methods.

#### Information on side effects and their management, return visits and switching

Only in 10 visits to facilities did providers spontaneously talk about side effects of the method(s) they suggested. Often, MCs had to probe to get information about potential problems with a method. MCs reported that even when providers talked about side effects, they shared only generalised information and rarely advised on follow-up or on the management of side effects.

“She suggested IUCD to me. When I asked if it would cause any problems, she said of course there would be some side effects. She neither said anything else nor gave me a chance to ask any questions.” *(Public facility, Bihar, ID57)*

In only 19 visits, providers advised MCs to come back later if they were unable to decide on a method at that moment. They were given the option to go home and discuss with their husbands and family before deciding. In only eight visits, providers informed MCs that they could switch to other methods later if they were unsatisfied with the method they chose during the visit.

### Quality of interpersonal relations

In this section, the findings related to the quality of interpersonal relations i.e. whether privacy and confidentiality were maintained by the provider, providers’ attitudes towards the client, and the duration of the interaction, are discussed.

#### Ensuring audio and visual privacy and confidentiality during the interaction

Analysis revealed that breach of privacy was a major point of concern in public health facilities. For instance, privacy was maintained during client-provider interactions in only 8 out of 36 interactions with MCs in public health facilities, compared with 25 out of 40 interactions in private facilities. In general, the providers interacted with MCs in the presence of other patients or other providers. Several client-provider interactions were interrupted by other patients or providers who were present in the room.

“There were so many people around, and in front of all, the doctor asked me what information I needed from her. The ANM [auxiliary nurse midwife] and other women, present there, kept interrupting our conversation. They were also giving advice for all my concerns. It was weird to talk to the doctor in front of other people. The doctor did not even try to stop them; she was also listening to them.” *(Public facility, Bihar, ID42)*

Those MCs who reported that their privacy was maintained while interacting with the provider, were more likely to recommend the facility to friends and family.

#### Providers’ attitudes towards clients

Many MCs reported that providers talked to them in an irritated tone and were always in a hurry, especially providers in public health facilities where they had to deal with a large number of patients. Private facilities were less crowded and providers there were more approachable.

“She was not giving me any attention and was looking around when I was asking questions. She said Copper-T would be best for you according to me and seemed irritated. She kept complaining about the workload. I requested her to tell me about injections in detail, but she was not listening to me.” *(Public facility, Bihar, ID30)*

#### Time given by providers

Mystery clients reported that most of the providers did not give them enough time, talking to them for seven minutes on average. As compared to public health facilities, providers of private health facilities spent more time with MCs, with an average duration of nine minutes in private and five minutes in public health facilities. Providers did not encourage detailed conversation and often MCs had to probe further.

“Why are you here? [Doctor asked.] … . I said, ‘I want to ask about family planning method’ … [Doctor further asked] ‘Ok, how many children do you have?’ … .’I have one child’ [I replied.] … ‘When did you have your last mensuration?’ … ‘Last week’ … ‘Ok, then IUCD will be suitable for you’ … I asked her if this was the only method and to tell me how to use it and what would be the cost. Only then she talked about other methods and their use.” *(Public facility, Bihar, ID53)*

## Discussion

Findings from this study reveal that quality of Client-provider interaction was not satisfactory in the states of UP and Bihar. Instead of sharing information about the full range of available methods as a “basket-of-choice”, providers suggested one or two contraceptive methods based on the client’s age and parity. Yet the standard guidelines for counselling women on FP do not place any restrictions based on age, parity, or any other characteristics of women, except for medical eligibility.^31–33^ Providers’ own imposed restrictions based on women’s age and parity were consistent with findings from several other studies.^34,35^

In recent years, FP programmes in India have had a special focus on increasing access to FP services among young and low parity women. A cadre of providers was trained to counsel married and unmarried boys and girls between the ages of 10–19 years as part of the Adolescent Reproductive and Sexual Health (ARSH) strategy.^36^ Findings of this study indicate that despite recent efforts by the government, providers were still hesitant to provide/suggest methods to young and zero parity women. Often, the suggestions of providers to these women were consistent with the local community’s social norms rather than the standard FP guidelines. Providers appeared worried about possible infertility caused by contraceptive methods and the family’s reaction following any incidence of side-effect. Past research also found that young people had to deal with age and agency related constraints for accessing sexual and reproductive health services.^37^ In many societies, including in India, proving fertility after marriage is very important and a deeply rooted social norm,^38–40^ which is also reflected in the findings of this study, as the majority of the providers cited were against suggesting many types of contraceptive use to newly married women. Marriage marks exposure to sexual activities for most young women in India,^41^ hence making it crucial to talk to them about contraceptives as well as STDs. However, providers missed this opportunity to counsel newly married women on HIV and STDs. Information on STDs and contraceptive methods would provide newly married clients with the necessary knowledge about their reproductive and sexual health choices and rights.

An informed choice is an integral part of QoC in FP, but our findings indicate that providers rarely had in-depth conversations with women. They immediately suggested just one or two methods depending on the clients’ characteristics or need; for example, female sterilisation for limiting, IUCD for spacing, or condoms for newly married women. Few MCs were asked about their preferred method or medical and contraceptive history. One recent study from India conducted in the same geographical areas found similar results from client exit interviews. The study highlighted that only 28% of FP clients were asked about their preference, told about at least one other method, or felt no pressure to accept a method and received method choice.^20^ Conversations about the side effects of the recommended method and their management or about follow-up care and options for method switching were extremely rare. Our findings that clients were not involved in the method selection indicate that the decision-making power of the client was minimal. Providers mostly suggested the methods that they thought appropriate for the clients.

Factors such as waiting time, privacy, provider’s tone and behaviour, availability of method choice and information about side effects, help to frame women’s perception on the quality of FP services.^9,42^ Our study showed very few providers considered these components during their interactions with clients. Clients feel more comfortable sharing personal issues when they are alone with providers.^43^ Lack of privacy and confidentiality are known to impact clients’ perception of whether they are satisfied with the services and these aspects are important for ensuring human rights are protected while providing FP services.

This study also allowed us to compare the quality of FP services between public and private facilities. Although there were not any prominent differences in suggestions made by providers, private facilities were better in terms of shorter waiting time to meet doctors, better privacy, tone and behaviour of the providers, and longer consultation time. One of the reasons behind this could be the heavier patient burden in public health facilities compared to private facilities.

It may be noted that the doctors with whom MCs interacted were also responsible for patients with other more serious health/medical conditions. MCs reported that providers seemed in a hurry while talking to them due to a heavy patient burden. They seemed to have a notion that it was not their job and they were only responsible for providing methods, but not the detailed information. There may be women who have not been contacted by any frontline health worker (FLW) or do not trust FLWs and contact a doctor directly for information and advice. Such women would remain unserved if doctors did not consider providing information as part of their job.

To reduce the burden on facility-based providers, the Government of India has approved the posting of one FP counsellor at each facility. These counsellors are responsible for providing comprehensive information on available FP services before clients make their decision on the method of choice. However, the availability of FP counsellors is still not universal in the health facilities of UP and Bihar.

## Limitations

When interpreting findings based on the experiences of MCs, it should be kept in mind that they are not the actual clients. MCs are trained professionals with a better understanding of quality services and may tend to be too critical in their assessments of the service quality or their satisfaction with the services. However, it can also be argued that since actual clients do not have much knowledge about their rights and what services they are entitled to receive, their expectations can be too low, and hence it would not be an accurate judgment of the QoC in services. The tone in which providers interact is also very critical in assessing the quality of the interaction but the study was not designed to capture these aspects.

## Conclusions

Emphasis on the quality of client-provider interaction regarding the provision of FP services would be helpful for ongoing programmes and in achieving goals. Findings indicate that providers had themselves imposed restrictions based on women’s age and parity, their suggestions were often influenced by local social norms, and information was rarely provided on side effects of the method and follow-up. Such learnings could be used as a key strategy in tackling two critical problems of FP practice in India: skewed method mix and high discontinuation rates. In view of these findings, it is recommended that the training given to all cadres of providers – from medical staff to FLWs – who provide FP services or advice/counselling, should be strengthened on all domains of QoC (information exchange, informed choice, clients’ concern and privacy). Training should also include how to counsel clients against the prevailing social norms of the community and especially on counselling zero parity women on delaying their first birth. Given the misconceptions among providers about side effects from certain contraceptive methods, future research could be aimed at investigating why providers promote certain methods in certain areas, from where these opinions stem, and what could be done to rectify this situation.

## Data Availability

The data that support the findings of this study are available upon reasonable request to the authors.

## References

[1] Conference on Human Rights. United Nations’ International Conference on Human Rights, at Tehran, Iran; 1968.

[2] ICPD. International Conference on Population and Development, at Cairo, Egypt; 1994.

[3] IPPF. International Planned Parenthood Federation (IPPF). Charter on Sexual and Reproductive Rights. IPPF; 1996. 10.1089/jwh.1.1999.8.459 10839699

[4] UNFPA. State of world population 2012. By choice, not by chance. Family Planning, Human Rights and Development; 2012.

[5] FP2020. Family planning 2020: rights and empowerment principles for family planning. Washington (DC): Rights and Empowerment Working Group; 2014.

[6] Hardee K, Kumar J, Newman K, et al. Voluntary, human rights-based family planning: a conceptual framework. Stud Fam Plann. 2014;45(1):1–18. doi: 10.1111/j.1728-4465.2014.00373.x 24615572

[7] Kumar J, Hardee K. Rights-based family planning: 10 resources to guide programming. Resource guide. Washington (DC): Population Council, The Evidence Project; 2015.

[8] Abdel-Tawab N, Roter D. The relevance of client-centered communication to family planning settings in developing countries: lessons from the Egyptian experience. Soc Sci Med. 2002;54(9):1357–1368. doi: 10.1016/S0277-9536(01)00101-0 12058852

[9] Jain AK, Hardee K. Revising the FP quality of care framework in the context of rights-based family planning. Stud Fam Plann. 2018;49(2):171–179. doi: 10.1111/sifp.12052 29708277

[10] Blanc AK, Curtis SL, Croft TN. Monitoring contraceptive continuation: links to fertility outcomes and quality of care. Stud Fam Plann. 2002;33:127–140. doi: 10.1111/j.1728-4465.2002.00127.x 12132634

[11] Sanogo D, RamaRao S, Jones H, et al. Improving quality of care and use of contraceptives in Senegal. Afr J Reprod Health. 2003;7:57–73. doi: 10.2307/3583214 14677301

[12] Jain A, Aruldas K, Tobey E, et al. Adding a Question about method switching to the method information index Is a better Predictor of contraceptive continuation. Glob Health: Sci Pract. 2019;7(2):289–299. 31249024 10.9745/GHSP-D-19-00028 PMC6641810

[13] World Health Organization Department of Reproductive Health and Research (WHO/RHR) and Johns Hopkins Bloomberg School of Public Health/Center for Communication Programs (CCP), Knowledge for Health Project. Family Planning: A Global Handbook for Providers (2018 update). Baltimore and Geneva: CCP and WHO, 2018. Available from: https://www.fphandbook.org/sites/default/files/global-handbook-2018-full-web.pdf

[14] Delbanco TL, Daley J. Through the patients’ eyes: strategies toward more successful contraception. Obstet Gynecol. 1996;88(3 Suppl):41S–47S. doi: 10.1016/0029-7844(96)00243-8 8752227

[15] Bruce J. Fundamental elements of the quality of care: a simple framework. Stud Fam Plann. 1990;21(2):61–91. doi: 10.2307/1966669 2191476

[16] Raymundo CM, Cruz GT. FP client-worker interaction as an ingredient of quality of care. Philipp Popul J. 1993;9(1–4):56–73. 12320233

[17] Harris S, Reichenbach L, Hardee K. Measuring and monitoring quality of care in family planning: are we ignoring negative experiences? Open Access J Contracept. 2016;7:97–108. 29386941 10.2147/OAJC.S101281 PMC5683163

[18] Hutchinson PL, Do M, Agha S. Measuring client satisfaction and the quality of family planning services: a comparative analysis of public and private health facilities in Tanzania, Kenya and Ghana. BMC Health Serv Res. 2011;11:203. doi: 10.1186/1472-6963-11-203 21864335 PMC3224259

[19] Tessema GA, Mahmood MA, Gomersall JS, et al. Client and facility level determinants of quality of care in family planning services in Ethiopia: multilevel modelling. PLoS One. 2017;12(6):e0179167. doi: 10.1371/journal.pone.0179167 28622376 PMC5473535

[20] Mozumdar A, Gautam V, Gautam A, et al. Choice of contraceptive methods in public and private facilities in rural India. BMC Health Serv Res. 2019;19(1):421. doi: 10.1186/s12913-019-4249-0 31238935 PMC6593496

[21] Dey A, Shakya HB, Chandurkar D, et al. Discordance in self-report and observation data on mistreatment of women by providers during childbirth in Uttar Pradesh, India. Reprod Health. 2017;14:149. doi: 10.1186/s12978-017-0409-z 29141640 PMC5688759

[22] Singh A, Chhugani M, Jame MM. Direct observation on respectful maternity care in India: a cross sectional study on health professionals of three different health facilities in New Delhi. Int J Sci Res.. 2016;7(5):821–825.

[23] Sugunadevi G. Quality of antenatal care services at subcentres: an infrastructure, process and outcome evaluation in a district in Tamil Nadu. Int J Community Med Public Health. 2017;4(11):4071–4077. doi: 10.18203/2394-6040.ijcmph20174647

[24] Rana MJ, Jain AK. Do Indian women receive adequate information about contraception? J Biosoc Sci. 2019;52(3):338–352. doi: 10.1017/S0021932019000488 31328714

[25] Naik S, Telma S, Lundgren R. Options of maintaining quality family planning counselling: Strategies for refresher training. Int J Qual Health Care. 2010;22(2):145–150. doi: 10.1093/intqhc/mzp062 20123698

[26] León FR, Lundgren R, Huapaya A, et al. Challenging the courtesy bias interpretation of favorable clients’ perceptions of family planning delivery. Eval Rev. 2007;31(1):24–42. doi: 10.1177/0193841X06289044 17259574

[27] Tumlinson K, Speizer IS, Curtis SL, et al. Accuracy of standard measures of family planning service quality: findings from the simulated client method. Stud Fam Plann. 2014;45(4):443–470. doi: 10.1111/j.1728-4465.2014.00007.x 25469929 PMC4258897

[28] International Institute for Population Sciences (IIPS) and ICF. National Family Health Survey (NFHS-4), 2015-16: India. Mumbai:IIPS. 2017.

[29] Braun V, Clarke V. Successful qualitative research: a practical guide for beginners. London: Sage; 2013.

[30] Guest G, Bunce A, Johnson L. How many interviews are enough?: An experiment with data saturation and variability. Field Methods. 2006;18:59–82. doi: 10.1177/1525822X05279903

[31] WHO. Family planning – a global handbook for providers 2018 edition; 2018.

[32] WHO. Decision-making tool for family planning clients and providers – a resource for high-quality counselling. World Health Organization. Department of Reproductive Health and Research; 2005.

[33] NRHM. Induction training module for ASHAs: A consolidated version of modules 1 to 5 for newly selected ASHAs. New Delhi: National Rural Health Mission. Ministry of Health & Family Welfare; 2016.

[34] Schwandt HM, Speizer IS, Corroon M. Contraceptive service provider imposed restrictions to contraceptive access in Urban Nigeria. BMC Health Serv Res. 2017;17(278):1–9. 28403858 10.1186/s12913-017-2233-0 PMC5389090

[35] Calhoun LM, Speizer IS, Rimal R, et al. Provider imposed restrictions to clients’ access to family planning in urban Uttar Pradesh, India: a mixed methods study. BMC Health Serv Res. 2013;13(532):1472–6963. (Electronic). 10.1186/1472-6963-13-532 PMC3879325 24365015

[36] NRHM. Implementation guide on RCH II ARSH strategy: for state and district programme managers. New Delhi Government of India, Ministry of Health & Family Welfare; 2006.

[37] Mchome Z, Richards E, Nnko S, et al. A ‘mystery client’ evaluation of adolescent sexual and reproductive health services in health facilities from two regions in Tanzania. PLoS ONE. 2015;10(3):1–11. doi: 10.1371/journal.pone.0120822 PMC4372447 25803689

[38] Eyayou Y, Berhane Y, Zerihun L. Socio-cultural factors in decisions related to fertility in remotely located communities: the case of the Suri ethnic group. Ethiop J Health Dev. 2004;18(3):171–174.

[39] Stanback J, Twumbah KA. Why do family planning providers restrict access to services? An examination in Ghana. Int Fam Plan Perspect. 2001;27(1):37–41. doi: 10.2307/2673804

[40] Lewis N. Quality of care in family planning service delivery in Kenya: clients’ and providers’ perspective. Nairobi: Population Council, Africa Operations Research and Technical Assistance Project; 1995.

[41] Santhya KG, Jejeebhoy SJ. Early marriage and HIV/AIDS: risk factors among young women in India. Econ Polit Wkly. 2007;42(14):1291–1297.

[42] Keesara SR, Juma PA, Harper CC. Why do women choose private over public facilities for family planning services? A qualitative study of post-partum women in an informal urban settlement in Kenya. BMC Health Ser Res. 2015;15:335. doi: 10.1186/s12913-015-0997-7 PMC4545906 26290181

[43] Gilson L, Magomi M, Mkangaa E. The structural quality of Tanzanian primary health facilities. Bull World Health Organ. 1995;73(1):105–114. 7704920 PMC2486583

